# Network structure analysis of depression, anxiety, and stress symptoms among college students: identification of central and bridge symptoms

**DOI:** 10.3389/fpsyt.2026.1811674

**Published:** 2026-05-26

**Authors:** Bochuan Zhao, Huiwen Zhu, Yuqi Su, Junji Chen, Haining Tu

**Affiliations:** 1 School of Tourism, Physical Education and Health, Guilin University, Guilin, Guangxi, China; 2 School of Outdoor Sports, Guilin Tourism University, Guilin, Guangxi, China; 3 Student Affairs Office, Guilin No. 2 Technical School, Guilin, China

**Keywords:** bridge symptoms, central symptoms, college students, mental health, network analysis

## Abstract

**Objective:**

Mental health problems are increasingly prevalent among college students, with depression, anxiety, and stress symptoms frequently co-occurring and mutually influencing one another. This study employed network analysis to examine the interrelationships among depression, anxiety, and stress symptoms in Chinese college students, aiming to identify potential targets to generate hypotheses for future precision-based interventions.

**Methods:**

A cross-sectional survey was conducted among college students at a Chinese university using the Depression Anxiety Stress Scale-21 (DASS-21). A Gaussian Graphical Model (GGM) combined with the graphical Least Absolute Shrinkage and Selection Operator (graphical LASSO) was used to construct the symptom network. Expected Influence (EI) was computed to identify central symptoms, and Bridge Expected Influence (Bridge EI) was calculated to identify bridge symptoms connecting different dimensions. Network structure visualization was also performed.

**Results:**

The symptom network revealed extensive positive connections among the 21 symptom nodes, with notable variation in edge strength. Central symptom analysis indicated that A15 (feeling of panic, EI = 2.06), S12 (difficulty relaxing, EI = 1.42), and A20 (feeling scared without reason, EI = 1.16) exhibited the highest expected influence. Bridge symptom analysis revealed that S8 (nervous tension, Bridge EI Z = 2.12), A15 (feeling of panic, Bridge EI Z = 1.70), and D13 (feeling downhearted and blue, Bridge EI Z = 1.51) were the key bridges connecting the depression, anxiety, and stress dimensions. Notably, A15 and S8 simultaneously ranked among the top central and bridge symptoms, indicating that these symptoms are identified as key nodes in both overall network activation and cross-dimensional symptom propagation.

**Conclusion:**

Feeling of panic, nervous tension, and difficulty relaxing are the central symptoms of mental health problems among college students, while nervous tension and feeling of panic simultaneously serve as bridge symptoms connecting different psychological problem dimensions. These symptoms represent theoretically-motivated candidate targets for future research. The findings support the application of transdiagnostic intervention strategies in college student mental health services.

## 1 Introduction

Mental health problems have become increasingly prevalent among college students, emerging as a focal point of global public health concern. The World Health Organization’s World Mental Health International College Student Survey project found that approximately one-third of college students reported at least one common DSM-IV disorder (1). Recent large-scale epidemiological surveys among Chinese college students have revealed detection rates of 9.8% for depression and 15.5% for anxiety, with a comorbidity rate of approximately 6.5% (2). These mental health problems not only impair academic performance and interpersonal relationships but are also closely associated with elevated dropout rates, substance abuse, and increased suicide risk (3, 4). Qualitative research has similarly revealed that sleep difficulties, anxiety, and stress are the three primary mental health challenges faced by Chinese college students in the post-pandemic era (5). Therefore, a deeper understanding of the internal structural relationships among depression, anxiety, and stress symptoms in the college student population holds significant theoretical and practical implications.

Traditionally, depression, anxiety, and stress have been conceptualized as independent constructs driven by their respective latent etiologies, with researchers typically employing latent variable models for separate assessment and intervention. However, extensive empirical evidence indicates that these psychological problems exhibit high comorbidity and complex bidirectional risk relationships. A longitudinal meta-analysis by Jacobson and Newman (2017) found that anxiety and depression serve as bidirectional risk factors for each other, with anxiety predicting future onset of depression and depression similarly predicting future exacerbation of anxiety (6). This high comorbidity phenomenon poses challenges to traditional categorical diagnostic approaches and single-disorder-oriented intervention strategies (7).

In recent years, network analysis has provided a novel theoretical perspective and analytical framework for understanding the complex relationships among psychological symptoms. Unlike traditional latent variable models (LVMs) that treat symptoms as passive indicators of underlying etiologies, network theory conceptualizes symptoms as active elements that exert mutual causal influences, ultimately forming self-sustaining vicious cycle networks (8, 9). This fundamental theoretical distinction enables network analysis to address scientific questions that LVMs cannot. First, while LVMs provide construct-level summaries, network analysis directly quantifies each symptom’s unique influence through centrality metrics, identifying which symptoms drive overall network activation (10, 11). Second, the mechanism of comorbidity, namely why depression, anxiety, and stress so frequently co-occur, is left unexplained by correlated latent factors in an LVM; instead, bridge centrality analysis explicitly identifies the symptom-level transmission pathways connecting different disorder clusters (12). Third, while previous DASS-21 network analyses have used large international samples (13), they have not been conducted in the specific cultural and developmental context of Chinese college students, nor have they simultaneously examined the overlap between central and bridge symptoms. Therefore, the present study was designed to fill these methodological and contextual gaps.

In symptom network research, centrality indices are essential tools for identifying central symptoms. Expected Influence (EI), proposed by Robinaugh et al. (2016) (14) in a bereaved sample with complicated grief, comprehensively accounts for both the direction and weight of edges and is considered a reliable indicator for evaluating the overall influence of a symptom within a network. Simulation studies have demonstrated that nodes with high expected influence exert a stronger impact on overall network activation, suggesting they may be important candidates for further investigation in intervention studies (15). Furthermore, the Bridge Centrality index proposed by Jones et al. (2021) can identify bridge symptoms connecting different symptom clusters (e.g., the depression cluster and the anxiety cluster) (12). It has been hypothesized that identifying and intervening on these bridge nodes may be a promising strategy for preventing the spread of comorbidity (12), a premise that has received preliminary support from simulation-based evidence (15). However, direct empirical testing through clinical trials remains needed to confirm these effects. These bridge symptoms play a critical role in the development and maintenance of psychological comorbidity, and intervening on these nodes is hypothesized to disrupt the potential cascading propagation of symptoms across dimensions.

Previous studies have applied network analysis to the field of college student mental health in various cultural settings. For instance, Tao et al. (2022) conducted a network analysis of depression, anxiety, and sleep disturbance symptoms among Chinese college students during the COVID-19 pandemic, identifying panic and alertness as the most central symptoms in the network (16). Wang et al. (2024) employed Bayesian network analysis to examine bridge relationships and potential causal pathways between depression and anxiety symptoms among a Chinese sample of first-year college students (17). Alvarenga et al. (2024) performed a network analysis of the DASS-21 in a Brazilian sample, revealing novel factors beyond the traditional three-factor structure (18). However, research on the DASS-21 symptom network among Chinese college students remains limited, particularly lacking comprehensive analyses that simultaneously identify both central and bridge symptoms.

The Depression Anxiety Stress Scale-21 (DASS-21) is a well-established instrument for assessing negative emotional states, comprising three subscales—depression, anxiety, and stress—with seven items each (19). This scale has demonstrated sound psychometric properties in Chinese populations (20), and its three-dimensional structure provides an ideal framework for exploring cross-dimensional symptom interactions. Van den Bergh et al. (2021) conducted a symptom network study of the DASS-21 using a large international sample (N = 11,647; majority Western, including 25% Malaysian), finding that panic, worry, worthlessness, hopelessness, and meaninglessness of life play key roles in depression–anxiety interactions (13). Given that Chinese college students face unique cultural contexts and stressors—including intense academic competition, employment pressure, and family expectations (5)—it is necessary to conduct DASS-21 symptom network research specifically within the Chinese college student population.

Growing evidence from Asian contexts specifically suggests that anxiety, and panic symptoms in particular, may occupy a structurally unique and clinically salient position in symptom networks compared to their role in Western samples. Park et al. (2020) conducted network analyses of depressive symptom profiles in Asian patients across six countries (China, Hong Kong, Japan, Korea, Taiwan, Singapore, etc), finding that anxiety-related symptoms consistently emerged as highly influential nodes within depressive disorder networks (21). Sönmez et al. (2025), in a study of common mental disorders in Indian public healthcare settings, found that panic was the most central symptom in a unified network of depression, anxiety, and somatic symptoms, and this finding was particularly pronounced among patients from lower socioeconomic backgrounds (22). These converging findings suggest that panic may function as a distinctive feature of distress expression and symptom organization in Asian populations, potentially reflecting culturally specific patterns of interoceptive sensitivity, stigma avoidance, or culturally normative interpretations of arousal states. The present study extends this line of inquiry to a Chinese college student population, a group with its own culturally specific stressors that has not previously been examined through this lens.

Based on the above background, the present study aimed to (1): construct the DASS-21 symptom network structure among Chinese college students (2); identify central symptoms within the network using expected influence (3); identify bridge symptoms connecting the depression, anxiety, and stress dimensions using bridge expected influence (4); examine the overlap patterns between central and bridge symptoms; and (5) generate preliminary hypotheses for precision-based mental health interventions.

## 2 Methods

### 2.1 Participants

A stratified cluster sampling method was employed to recruit participants from four universities in Guangxi Province, China (Guangxi Normal University, Guilin University of Information Science and Technology, Guilin University, and Guilin University of Information Engineering). Inclusion criteria were (1): full-time enrolled college students (2); provision of informed consent to participate. Exclusion criteria were (1): excessively short questionnaire completion time (2); presence of clearly patterned response styles. The final valid sample comprised 2,291 participants, including 609 males (26.6%) and 1,682 females (73.4%), with ages ranging from 18 to 25 years (M = 20.24, SD = 0.96). The grade distribution was as follows: 1,193 freshmen (52.1%), 1,075 sophomores (46.9%), 13 juniors (0.6%), and 10 seniors (0.4%). A total of 302 participants (13.2%) were only children, while 1,989 (86.8%) had siblings.

### 2.2 Instruments

The Depression Anxiety Stress Scale-21 (DASS-21) was administered for assessment (18). This scale comprises 21 items divided into three subscales: Depression subscale (D): Items 3 (D3: Nothing to look forward to), 5 (D5: Difficulty initiating activities), 10 (D10: Feeling hopeless), 13 (D13: Feeling downhearted and blue), 16 (D16: Lack of interest), 17 (D17: Feeling life is meaningless), and 21 (D21: Feeling worthless), assessing symptoms of hopelessness, anhedonia, and self-deprecation. Anxiety subscale (A): Items 2 (A2: Dryness of mouth), 4 (A4: Breathing difficulty), 7 (A7: Trembling), 9 (A9: Worry about embarrassing situations), 15 (A15: Feeling of panic), 19 (A19: Heart palpitations), and 20 (A20: Feeling scared without reason), assessing autonomic arousal, panic, and fear. Stress subscale (S): Items 1 (S1: Difficulty calming down), 6 (S6: Overreacting to situations), 8 (S8: Nervous tension), 11 (S11: Being easily irritated), 12 (S12: Difficulty relaxing), 14 (S14: Being easily impatient), and 18 (S18: Being easily agitated), assessing tension, irritability, and difficulty relaxing. Each item is rated on a 4-point Likert scale (0 = “Did not apply to me at all” to 3 = “Applied to me very much or most of the time”), with higher scores indicating greater symptom severity. The present study used the Chinese version of the DASS-21, which has demonstrated a stable three-factor structure and good internal consistency in Chinese populations (23).

### 2.3 Data collection procedure

Data were collected via an online questionnaire. All participants read the informed consent form prior to questionnaire completion and could proceed to respond only after understanding the research purpose, confidentiality principles, and principles of voluntary participation.

### 2.4 Statistical analysis

#### 2.4.1 Network estimation

A Gaussian Graphical Model (GGM) was employed to construct the symptom network. The GGM estimates conditional dependence relationships between nodes through partial correlation coefficients, controlling for the influence of all other nodes in the network, thereby revealing the direct associations between pairwise symptoms. Prior to network estimation, the distributional characteristics of all 21 items were examined; given the ordinal nature of the 4-point response scale and the observed non-normal distribution (skewness range: 0.76–2.72), polychoric correlations were computed as the basis for network estimation. This approach is more appropriate than Pearson-based correlations for ordinal data, as it better accounts for the categorical nature of the responses. To address the issue of false-positive connections in high-dimensional data, the graphical Least Absolute Shrinkage and Selection Operator (graphical LASSO) was applied for regularization, with the Extended Bayesian Information Criterion (EBIC) used to select the optimal tuning parameter (γ = 0.5). This approach compresses weak and spurious connections to zero through L1 penalization, generating a sparse and interpretable network structure that controls false positives while preserving meaningful connections.

#### 2.4.2 Identification of central symptoms

Expected Influence (EI) was calculated as the indicator for identifying central symptoms. EI is defined as the algebraic sum of all edge weights connected to a node, with positive edges contributing positive values and negative edges contributing negative values. In symptom networks predominantly characterized by positive connections, a high EI value indicates that the symptom has strong positive associations with other symptoms in the network, and activation of that symptom is hypothesized to be associated with potential cascading activation of other symptoms. EI is superior to traditional strength centrality in networks containing both positive and negative edges, as the latter, which employs absolute values, may overestimate the importance of nodes with numerous negative connections.

#### 2.4.3 Identification of bridge symptoms

Bridge Expected Influence (Bridge EI) was employed to identify bridge symptoms connecting different symptom dimensions (Jones et al., 2021). Bridge EI is defined as the algebraic sum of edge weights between a node and nodes belonging to other symptom clusters (communities). Based on the theoretical structure of the DASS-21, the 21 symptom nodes were assigned to three communities: depression (D3, D5, D10, D13, D16, D17, D21), anxiety (A2, A4, A7, A9, A15, A19, A20), and stress (S1, S6, S8, S11, S12, S14, S18). A high Bridge EI value indicates that the symptom plays a critical role in cross-dimensional symptom propagation. To facilitate comparison across different indices, raw Bridge EI values were standardized by converting them to Z-scores.

#### 2.4.4 Network visualization

The Fruchterman–Reingold algorithm (“spring” layout) was used for network visualization. In this layout, nodes with stronger connections tend to be positioned closer together, forming cluster structures. Edge thickness reflects the absolute value of the partial correlation coefficient (connection strength), with blue edges representing positive connections and red edges representing negative connections. Node shapes were used to differentiate symptom dimensions: circles represent depression symptoms (D), triangles represent anxiety symptoms (A), and squares represent stress symptoms (S).

#### 2.4.5 Software and packages

All analyses were conducted using R software (version 4.3.0). The primary packages used were: *psych* for descriptive statistics, *bootnet* for network estimation, *qgraph* for network visualization and centrality computation, *networktools* for bridge centrality computation, and *ggplot2* for graphical presentation.

## 3 Results

### 3.1 Descriptive statistics

The descriptive statistics and distributional characteristics of the items are presented in **Table 1**. All items showed positive skewness ranging from 0.76 to 2.72, justifying the use of polychoric correlations.

**Table 1:** Descriptive statistics and distributional characteristics of the DASS-21 items (N = 2,291).

Node	Symptom description	Mean	SD	Skewness	Kurtosis
Stress					
S1	Difficulty calming down	1.61	0.78	1.18	0.84
S6	Overreacting to situations	1.48	0.73	1.54	1.92
S8	Nervous tension	1.53	0.77	1.46	1.59
S11	Being easily irritated	1.55	0.77	1.33	1.14
S12	Difficulty relaxing	1.54	0.77	1.37	1.30
S14	Being easily impatient	1.68	0.85	1.09	0.37
S18	Being easily agitated	1.47	0.72	1.57	2.08
Anxiety					
A2	Dryness of mouth	1.84	0.86	0.78	-0.16
A4	Breathing difficulty	1.38	0.66	1.82	2.99
A7	Trembling	1.48	0.74	1.53	1.77
A9	Worry about embarrassing situations	1.90	0.94	0.76	-0.40
A15	Feeling of panic	1.47	0.72	1.53	1.91
A19	Heart palpitations	1.42	0.70	1.76	2.78
A20	Feeling scared without reason	1.36	0.67	1.97	3.58
Depression					
D3	Nothing to look forward to	1.51	0.73	1.45	1.72
D5	Difficulty initiating activities	1.65	0.83	1.16	0.63
D10	Feeling hopeless	1.52	0.78	1.48	1.53
D13	Feeling downhearted and blue	1.54	0.73	1.33	1.44
D16	Lack of interest	1.46	0.72	1.60	2.12
D17	Feeling life is meaningless	1.25	0.60	2.72	7.41
D21	Feeling worthless	1.32	0.64	2.23	4.91

SD, standard deviation. All items were scored on a 4-point Likert scale (0–3). Skewness values range from 0.76 to 2.72, indicating a positive skew across all symptoms.

### 3.1 Symptom network structure

**Figure 1:**
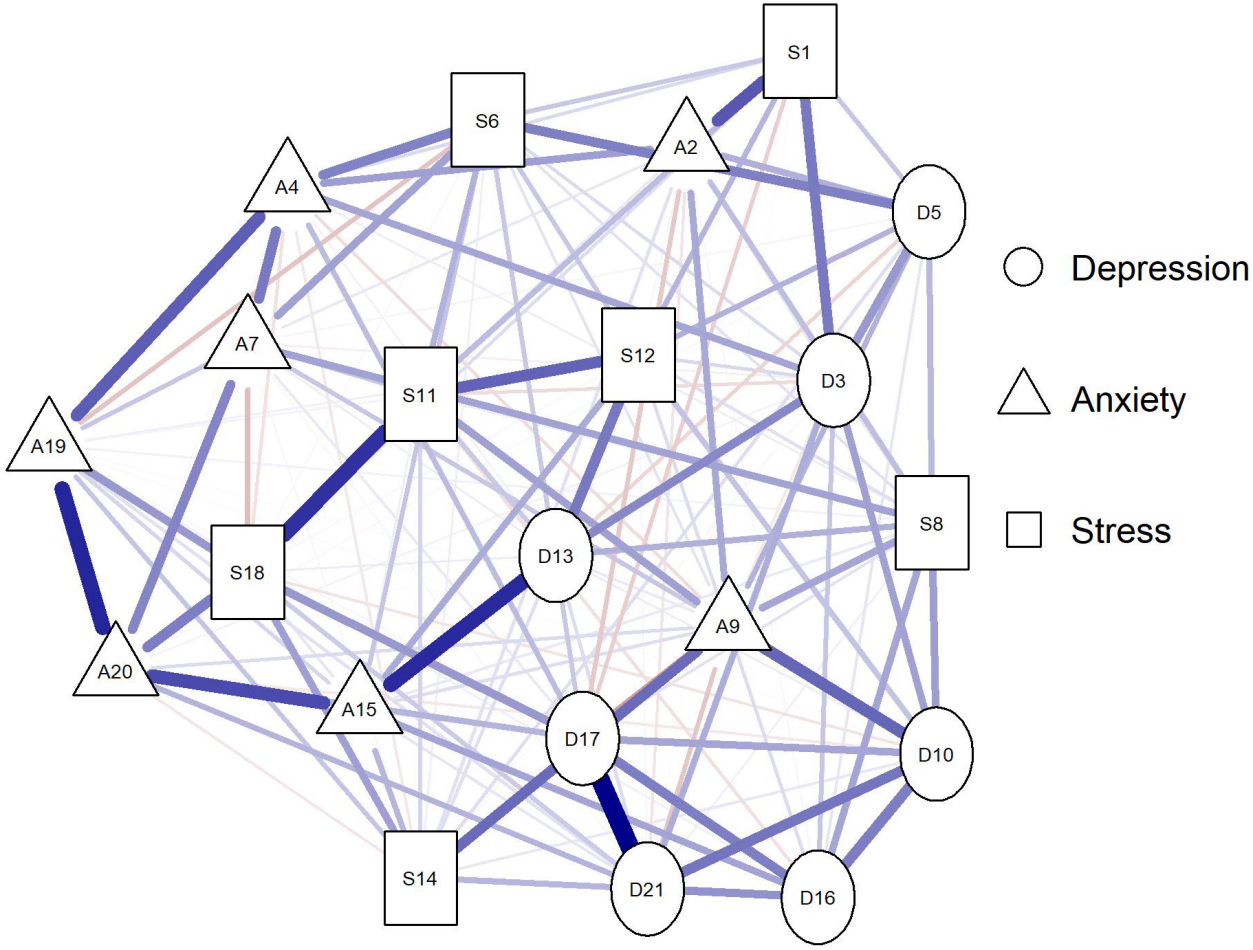
DASS-21 symptom network structure among college students. Nodes represent the 21 symptom items. Circles, depression symptoms (D), triangles, anxiety symptoms (A), squares, stress symptoms (S). Edge thickness represents the absolute value of partial correlation coefficients (connection strength); blue, positive connections; red, negative connections. Fruchterman–Reingold spring layout was applied, with closely connected nodes positioned nearer to each other.

**Figure 1** presents the DASS-21 symptom network structure among college students. The network revealed extensive positive connections (blue edges) among the 21 symptom nodes, with only a few negative connections (red edges). Visual inspection indicated that symptoms within the same dimension tended to cluster together: depression symptoms (circular nodes), anxiety symptoms (triangular nodes), and stress symptoms (square nodes) each formed relatively dense clusters. However, cross-dimensional connections were also notably present, particularly between stress-dimension symptoms and anxiety-dimension symptoms, indicating complex interrelationships among depression, anxiety, and stress symptoms.

The strongest within-dimension edges in the network included: a strong positive connection between D17 (life is meaningless) and D21 (feeling worthless; weight = 0.36), a strong positive connection between A19 (heart palpitations) and A20 (feeling scared without reason; weight = 0.304), and a strong positive connection between S11 (being easily irritated) and S18 (being easily agitated; weight = 0.289). These strong within-dimension connections reflect the high conditional dependence between theoretically closely related symptom pairs. Additionally, A15 (feeling of panic) exhibited a broad pattern of connections with multiple nodes across different dimensions, suggesting its hub-like position within the overall network. Notably, the cross-dimensional connection between D13 (feeling downhearted and blue) and A15 (feeling of panic; weight = 0.298) was among the strongest in the network, providing visual support for the subsequent bridge symptom analysis.

### 3.2 Central symptom analysis

**Figure 2:**
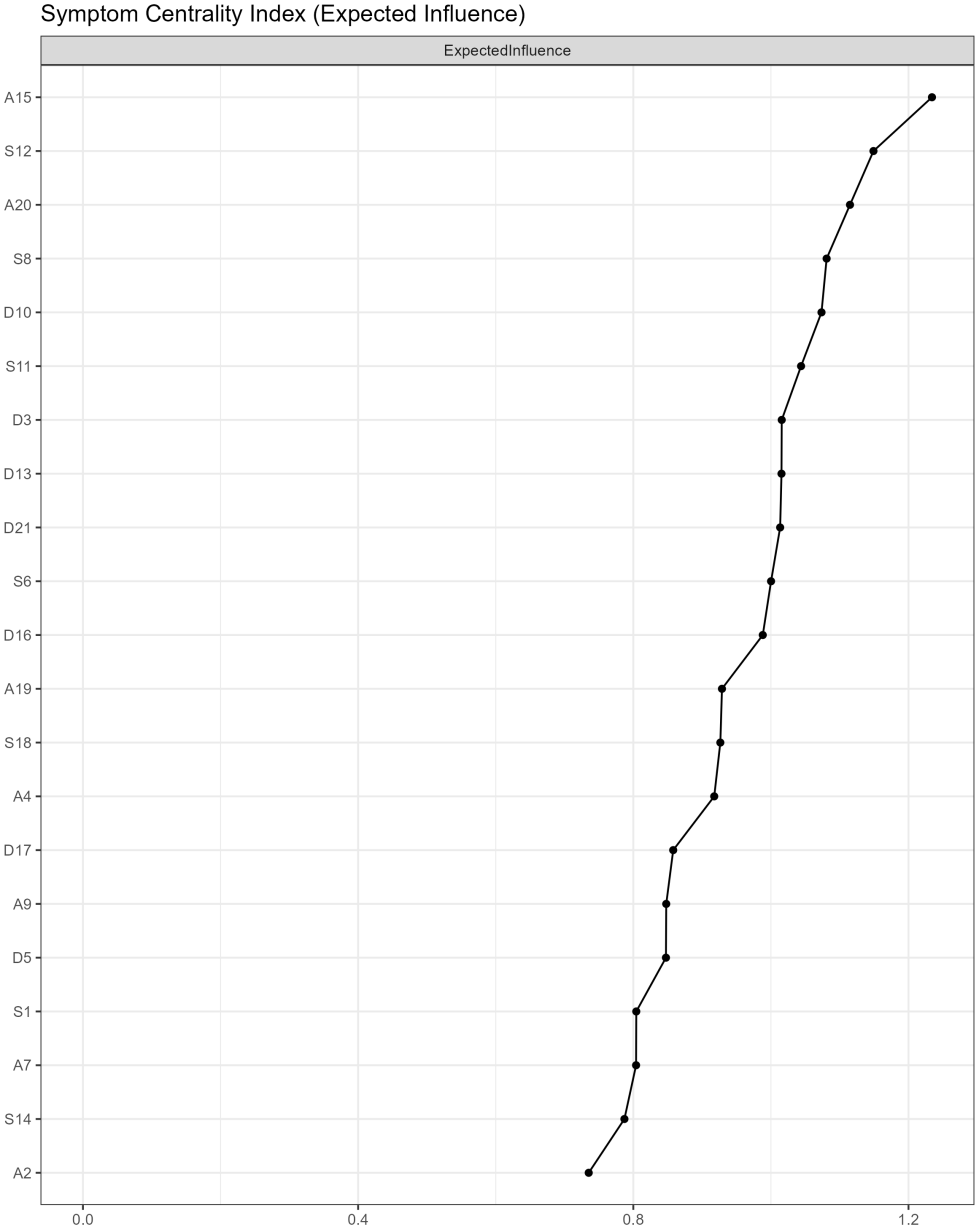
Symptom centrality index (expected influence). The horizontal axis represents Expected Influence values; the vertical axis represents symptom nodes, arranged in descending order of centrality values.

**Figure 2** and **Table 2** present the Expected Influence (EI) results for the 21 symptoms. The five symptoms with the highest expected influence were: A15 (feeling of panic, EI = 2.06), S12 (difficulty relaxing, EI = 1.42), A20 (feeling scared without reason, EI = 1.16), S8 (nervous tension, EI = 0.91), and D10 (feeling hopeless, EI = 0.84).

**Table 2:** Expected influence ranking of symptoms (top 5).

Rank	Node	Symptom description	Dimension	Expected Influence Z-score
1	A15	Feeling of panic	Anxiety	2.06
2	S12	Difficulty relaxing	Stress	1.42
3	A20	Feeling scared without reason	Anxiety	1.16
4	S8	Nervous tension	Stress	0.91
5	D10	Feeling hopeless	Depression	0.84

Notably, the central symptoms were distributed across all three dimensions, with A15 from the anxiety dimension exhibiting a markedly higher EI value (2.06), substantially exceeding the second-ranked S12 (1.42). The anxiety dimension contributed two central symptoms (A15 and A20), the stress dimension contributed two (S12 and S8), and the depression dimension contributed one (D10). This cross-dimensional distribution pattern suggests that mental health problems among college students are not dominated by a single dimension but rather involve complex interactions among symptoms related to anxious arousal, chronic tension, and low mood, with feeling of panic and unexplained fear occupying central positions.

### 3.3 Bridge symptom analysis

**Figure 3:**
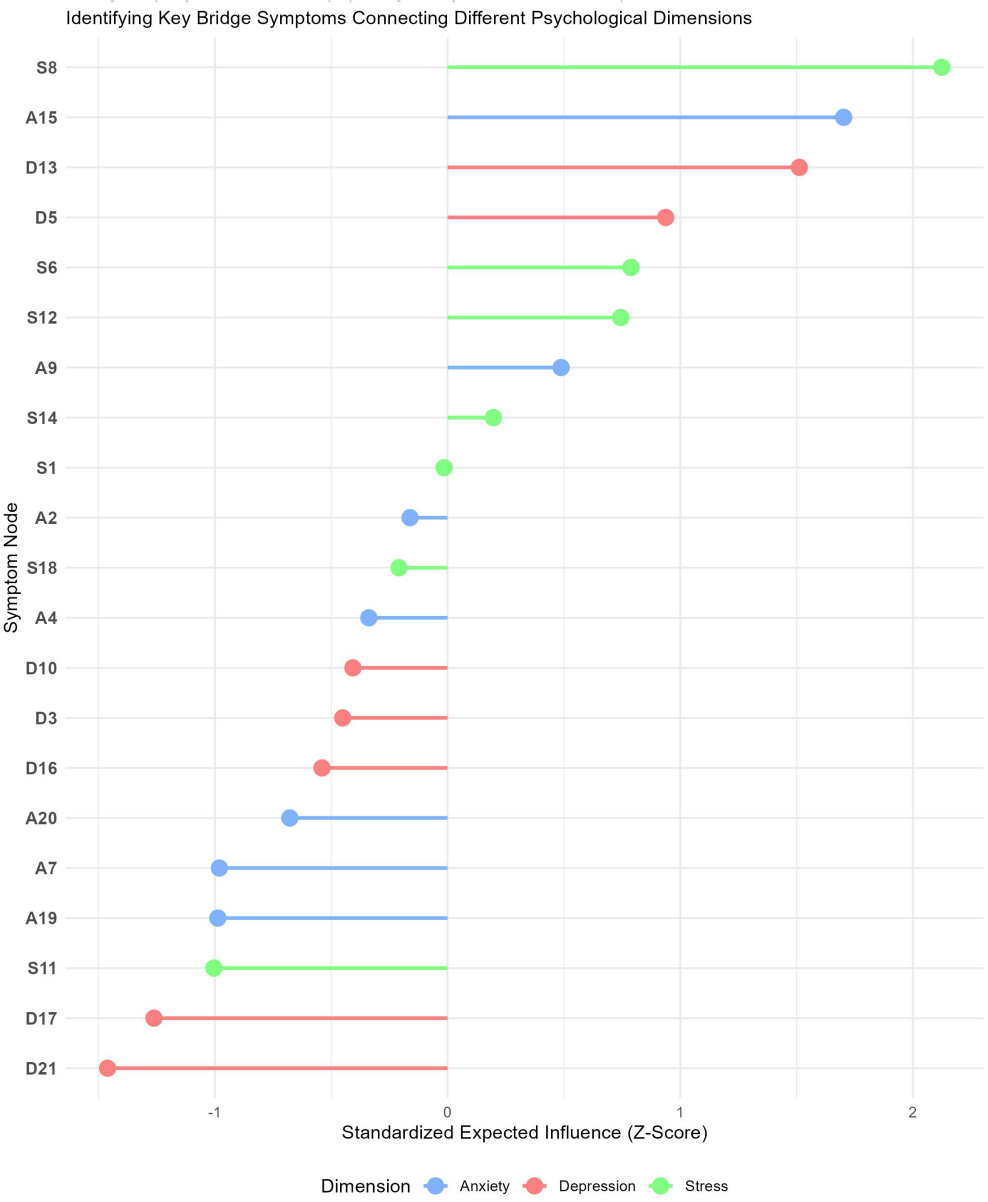
Bridge symptom centrality. The horizontal axis represents standardized Bridge Expected Influence (Z-scores); the vertical axis represents symptom nodes. Colors differentiate dimensions: red, depression; blue, anxiety; green, stress.

**Figure 3** and **Table 3** present the Bridge Expected Influence (Bridge EI) analysis results. Based on raw Bridge EI values and their standardized Z-scores, the five symptoms with the highest bridge centrality were: S8 (nervous tension, raw Bridge EI = 0.98, Z = 2.12), A15 (feeling of panic, raw Bridge EI = 0.88, Z = 1.70), D13 (feeling downhearted and blue, raw Bridge EI = 0.84, Z = 1.51), D5 (difficulty initiating activities, raw Bridge EI = 0.70, Z = 0.94), and S6 (overreacting to situations, raw Bridge EI = 0.66, Z = 0.79).

**Table 3:** Bridge symptom ranking (top 5).

Rank	Node	Symptom description	Dimension	Bridge EI (Raw)	Z-score
1	S8	Nervous tension	Stress	0.98	2.12
2	A15	Feeling of panic	Anxiety	0.88	1.70
3	D13	Feeling downhearted and blue	Depression	0.84	1.51
4	D5	Difficulty initiating activities	Depression	0.70	0.94
5	S6	Overreacting to situations	Stress	0.66	0.79

Bridge symptoms were distributed across all three dimensions, with S8 from the stress dimension exhibiting the highest bridge centrality (Z = 2.12), indicating that “nervous tension” is the most critical bridge connecting the stress symptom cluster with the anxiety and depression symptom clusters. A15 from the anxiety dimension (feeling of panic, Z = 1.70) and D13 from the depression dimension (feeling downhearted and blue, Z = 1.51) also demonstrated substantial bridge effects. The fact that the top three bridge symptoms each originated from a different dimension indicates that cross-dimensional symptom propagation involves multiple pathways.

### 3.4 Overlap between central and bridge symptoms

**Table 4** presents a comprehensive comparison of the central and bridge symptom rankings. A15 (feeling of panic), S8 (nervous tension) simultaneously appeared in the top five of both rankings, indicating that these two symptoms possess the dual characteristics of “high network influence” and “high cross-dimensional propagation capacity.”

**Table 4:** Comparison of central and bridge symptom rankings.

Node	Symptom description	Dimension	EI rank	Bridge EI rank	Dual role
A15	Feeling of panic	Anxiety	1	2	✓
S12	Difficulty relaxing	Stress	2	—	
A20	Feeling scared without reason	Anxiety	3	—	
S8	Nervous tension	Stress	4	1	✓
D13	Feeling downhearted and blue	Depression	—	3	
D5	Difficulty initiating activities	Depression	—	4	
S6	Overreacting to situations	Stress	—	5	

This dual role implies that interventions targeting A15 and S8 are hypothesized to be associated with reduce overall network activation levels and may disrupt symptom propagation across the depression, anxiety, and stress dimensions; therefore, these symptoms could be considered potential candidates for targeted interventions in future longitudinal research.

## 4 Discussion

The present study employed network analysis to systematically explore the network structure of DASS-21 symptoms among Chinese college students, successfully identifying central symptoms and bridge symptoms, and revealing significant overlap between these two categories. The following discussion addresses the overall characteristics of the symptom network, the clinical significance of central symptoms, bridge symptoms and comorbidity mechanisms, the intervention implications of central–bridge overlap, and research limitations.

### 4.1 Overall characteristics of the symptom network

The DASS-21 symptom network constructed in the present study exhibited a topological structure predominantly characterized by extensive positive connections, which is consistent with the core hypothesis of network theory that psychological symptoms mutually activate one another (9). Within-dimension connection strength was generally higher than cross-dimensional connections, reflecting the validity of the original three-factor structure of the DASS-21 (24). However, the widespread presence of cross-dimensional connections suggests that depression, anxiety, and stress do not function as mutually independent systems but rather interpenetrate and influence one another through specific symptoms. This finding is highly consistent with the DASS-21 network analysis conducted by Van den Bergh et al. (2021) based on a large international sample (N = 11,647), which similarly observed significant cross-cluster connections among the three symptom groups of depression, anxiety, and stress (20).

The strongest within-dimension connections in the network—D21 (worthlessness) and D17 (life is meaningless), A19 (heart palpitations) and A20 (feeling scared without reason), and S11 (being easily irritated) and S18 (being easily agitated)—revealed core symptom pairs within each dimension, providing valuable information for understanding the symptom dynamics within each psychological problem. Specifically, the strong connection between D21 and D17 reflects the close association between core cognitive symptoms of depression, consistent with the intimate link between negative self-evaluation and hopelessness emphasized by Beck’s cognitive theory (25). The strong connection between A19 and A20 reflects the co-occurrence pattern of physiological arousal and subjective fear experiences within anxiety symptoms (26). Notably, the strong connection between S11 and S18 reflects the “irritability” component of stress. In the context of Chinese college students, this may manifest as a heightened emotional reactivity and diminished patience under prolonged academic and competitive pressures.

### 4.2 Clinical significance of central symptoms

The present study found that A15 (feeling of panic) was the node with the highest expected influence in the DASS-21 symptom network among college students (EI = 2.06), substantially exceeding other symptoms. As a core feature of anxiety, feeling of panic involves intense somatic sensations (e.g., accelerated heartbeat, breathing difficulty) and catastrophic cognitive interpretations (e.g., “I am going to lose control”), and its prominent central position may reflect the potential core position of this symptom in activating and maintaining overall psychological distress. Specifically, its high centrality may reflect the theoretical role of this symptom in co-occurring with other symptoms through multiple channels: on one hand, through the physiological arousal pathway activating other anxiety symptoms, and on the other hand, through catastrophic interpretations triggering hopelessness and helplessness characteristic of depressive symptoms. Notably, A20 (feeling scared without reason, EI = 1.16) also emerged as the third most central symptom. The co-occurrence of high centrality in both A15 and A20 suggests that anxious arousal characterized by both acute autonomic surges and generalized subjective fear is a primary driver of distress in this population. Culturally, the prominence of A15 (感到恐慌, gǎn dào kǒng huāng) aligns with recent evidence from Asian samples (Sönmez et al., 2025), where panic symptoms function as a pivotal “warning sign” of systemic distress. This may reflect a culturally specific idiom where acute arousal overlaps with “intense tension,” a phenomenon particularly salient under the intense competition faced by Chinese students. This finding is consistent with previous network analysis studies using different scales and samples. Beard et al. (2016) found in a depression–anxiety network analysis of a psychiatric sample that panic-related symptoms were among the most influential nodes in the anxiety cluster (27). Tao et al. (2022) similarly identified panic (feeling afraid) as one of the most central symptoms in the anxiety–depression–sleep disturbance network among college students. Van den Bergh et al. (2021) also found that panic-related symptoms exhibited high influence within the DASS-21 network (20). From a developmental psychopathology perspective, panic experiences may maintain and amplify psychological distress through mechanisms of conditioned fear acquisition and interoceptive hypersensitivity.

S12 (difficulty relaxing, EI = 1.42) and S8 (nervous tension, EI = 0.91), as the two central symptoms from the stress dimension, reflect the persistent psychological tension and elevated arousal state among the college student population. Lovibond et al. (1995) noted during scale development that the DASS stress subscale assesses a chronic nonspecific arousal state, and this persistent tension may reflect the characteristic difficulty individuals experience in recovering from stress states (24). For college students, academic pressure, employment competition, and interpersonal relationship challenges constitute multiple chronic stressors, and sustained nervous tension and difficulty relaxing may serve as preconditions that trigger anxiety and depressive symptoms. Wang et al. (2023) similarly found that “difficulty relaxing” exhibited high expected influence in a Chinese community resident sample, further supporting the central position of stress symptoms within the psychological distress network (28).

D10 (feeling, hopeless EI = 0.84) represents a core cognitive feature of the depression dimension. Its prominent central position resonates with cognitive models of depression, which emphasize the critical roles of negative expectations and hopelessness in the development and maintenance of depression (25). Network analysis studies of depression have repeatedly found that sad mood and hopelessness are nodes with significant influence in depression symptom networks (29, 30).

### 4.3 Bridge ssymptoms and comorbidity mechanisms

The bridge symptom analysis represents one of the central findings of the present study. S8 (nervous tension) exhibited the highest bridge expected influence (Z = 2.12), indicating that this symptom is the most critical node for cross-dimensional symptom propagation. From a clinical perspective, “nervous tension” is a nonspecific high-arousal state situated at the intersection of stress, anxiety, and depression: it is both a direct response manifestation of stress and a driving force for anxiety symptoms (e.g., panic, excessive worry), while prolonged nervous tension can promote the development of depressive symptoms through the sustained depletion of an individual’s psychological resources (31, 32). This finding provides a symptom-level mechanistic explanation for understanding stress–anxiety–depression comorbidity, suggesting that “nervous tension” may serve as the central hub for transmission among the three negative emotional states. This resonates with non-Western clinical contexts where “tension” is a primary idiom of distress, often associated with traditional Chinese psychiatric concepts such as neurasthenia. Previous network analysis studies have similarly found that “nervous restlessness”-type symptoms serve as important bridges between anxiety and depression (16).

A15 (feeling of panic, Z = 1.70) simultaneously serves as both a central symptom (EI rank 1) and a bridge symptom (Bridge EI rank 2), and this dual role is particularly noteworthy. Panic experiences not only involve intense immediate anxiety but may also propagate to other dimensions through multiple pathways: avoidance behavior triggered by panic can lead to functional impairment, subsequently eliciting secondary depressive mood and feelings of frustration (33); repeated occurrences of panic experiences can also increase individuals’ perception of stress, exacerbating overall tension levels. Van den Bergh et al. (2021) similarly found in their DASS-21 network study that panic-related symptoms serve as important bridges connecting the anxiety cluster with other symptom clusters (20), and the social consequences of public panic may exacerbate feelings of worthlessness, subsequently leading to social withdrawal and other depressive manifestations. Therefore, feeling of panic may function as the origin or amplifier of multiple cross-dimensional propagation pathways, and its bridge role carries important clinical implications.

D13 (feeling downhearted and blue, Z = 1.51), as the highest-ranked bridge symptom from the depression dimension, may represent a key channel through which emotional distress disseminates to other dimensions. Tahmassian et al. (2011) found that self-efficacy (particularly emotional self-efficacy) was significantly negatively correlated with anxiety and worry symptoms (34), suggesting that sustained depressed mood may increase sensitivity to threatening stimuli by depleting individuals’ coping resources and self-efficacy, thereby activating anxiety and stress symptoms. Kaiser et al. (2021) conducted a depression–anxiety bridge symptom analysis in a large inpatient sample and found that sad mood and inability to control worry were important bridges connecting depression and anxiety; the present study further validated these findings in a subclinical college student population (7).

### 4.4 Theoretical and clinical implications of the overlap between central and bridge symptoms

The finding that A15 and S8 simultaneously exhibit high centrality and high bridge centrality carries important theoretical and practical implications. From the perspective of network theory, these symptoms not only possess the strongest activation capacity within the overall network but also serve as the most important transmitters between different symptom clusters, and can be regarded as “hub symptoms” that maintain comorbid states. From an intervention perspective, this implies that targeted treatment of these symptoms is hypothesized to be a more efficient strategy for generating hypotheses about symptom reduction.

### 4.5 Intervention implications

Based on the network analysis results presented above, the present study proposes the following tiered intervention recommendations. First, interventions targeting central symptoms represents a promising starting point for clinical investigation. Interoceptive exposure and cognitive reappraisal techniques from Cognitive Behavioral Therapy (CBT) can be applied to address feeling of panic (A15). Given that feeling of panic simultaneously serves as both a central and bridge symptom, successful alleviation of panic may produce cross-dimensional improvement effects. For nervous tension (S8) and difficulty relaxing (S12), techniques aimed at reducing physiological arousal—such as mindfulness meditation, progressive muscle relaxation, and relaxation training—can directly target these chronic high-arousal symptoms. Second, given the dual central–bridge roles of S8 and A15, prioritizing interventions for these symptoms may effectively disrupt cross-dimensional symptom propagation. For example, interventions targeting “nervous tension” (e.g., relaxation training) can not only directly alleviate stress symptoms but may also indirectly reduce anxiety and depression symptom levels by diminishing the transmission effects to the anxiety and depression clusters. Additionally, as the primary bridge symptom for depression, targeting “feeling downhearted and blue” (D13), behavioral activation therapy can effectively improve low mood by increasing positive behaviors and environmental rewards, thereby breaking the avoidance cycle characteristic of depression. Third, the present findings support the application of transdiagnostic intervention approaches. The cross-dimensional bridge symptoms and central–bridge overlap patterns identified in this study indicate that depression, anxiety, and stress are highly interconnected at the symptom level, supporting intervention strategies that target common vulnerability factors rather than individual disorders. Finally, for university mental health professionals, we recommend incorporating the central and bridge symptoms identified in this study into screening and assessment protocols as early warning indicators. When students report significant feelings of panic, persistent tension, or profound despondency, clinicians should be alert to the risk of cross-dimensional symptom spread and initiate targeted interventions promptly. The findings of this study should be interpreted as generating hypotheses rather than testing them. While cross-sectional networks cannot establish causal direction, the central and bridge symptoms identified, particularly A15 (feeling of panic), S8 (nervous tension), D10 (feeling hopeless), and D13 (feeling downhearted and blue), constitute empirically motivated “candidate targets” for future investigation. Identifying these targets is a necessary first step for developing culturally-tailored interventions in the Chinese context. In this specific developmental setting, “anxious arousal” (A15, A20) may play a uniquely influential role compared to Western samples, possibly reflecting the acute emotional reactivity triggered by intense competition. Future clinical trials or high-frequency EMA designs are needed to confirm whether targeting these specific “hub symptoms” produces superior outcomes in Chinese college students.

### 4.6 Strengths and limitations

The present study has several notable strengths. First, the Chinese cultural context of the sample constitutes a significant scientific strength. This research sit at the intersection of emerging Asian research suggesting that anxiety (21, 22), particularly “panic” symptoms, occupied a more central role than typically observed in Western community samples. Second, the use of the DASS-21 as a unified instrument avoids the problem of “spurious bridge symptoms” that can arise when combining distinct scales with overlapping items (e.g., PHQ-9 and GAD-7), thereby enhancing the methodological rigor of our bridge symptom analysis.

Several limitations of the present study warrant acknowledgment. First, the cross-sectional design precludes inference of causal relationships or temporal precedence among symptoms; the edges in the network reflect only conditional dependence relationships rather than causal effects. Future research could employ the Experience Sampling Method (ESM) to collect intensive time-series data and construct temporal and contemporaneous networks to elucidate dynamic causal relationships among symptoms. Second, the sample was drawn from a convenience sample within a single province of China, and the generalizability of the findings requires verification in multi-center, multi-regional samples. Third, the present study relied solely on self-report scale data without incorporating clinical diagnostic information; the findings are primarily applicable to subclinical populations rather than clinical samples. Fourth, although the DASS-21 has undergone thorough psychometric validation, its limited number of items may not fully capture all symptom manifestations of depression, anxiety, and stress. Future research could consider integrating multiple instruments to obtain a more comprehensive symptom network. A fifth limitation concerns the inferential scope of network centrality metrics. As emphasized by recent methodological critiques, highly central symptoms might function as “causal endpoints” rather than drivers, meaning that intervening on them might not lead to change in the rest of the network (35). Furthermore, the “fat hand” problem suggests that it is practically difficult to isolate and target a single symptom without affecting the broader system (36). Therefore, our findings should be understood as preliminary insights into co-occurrence patterns rather than as prescriptive clinical recommendations.

## 5 Conclusion

The present study employed network analysis to explore the network structure of DASS-21 symptoms among Chinese college students, systematically identifying central and bridge symptoms. The findings revealed that (1): feeling of panic (A15), difficulty relaxing (S12), and nervous tension (S8) are the central symptoms with the highest expected influence in the network, distributed across the anxiety and stress dimensions; (2) nervous tension (S8), feeling of panic (A15), and feeling downhearted and blue (D13) are the key bridge symptoms connecting the depression, anxiety, and stress dimensions, with each dimension contributing one critically important bridge; (3) A15 and S8 simultaneously exhibit high centrality and high bridge centrality and are identified as key potential targets that warrant further investigation in longitudinal and clinical designs. The findings provide empirical evidence for precision-based mental health interventions among college students, support the application of targeted treatment of key “hub symptoms” and transdiagnostic intervention strategies in university mental health services, and offer a new perspective for understanding the symptom-level mechanisms of depression–anxiety–stress comorbidity.

## Data Availability

The raw data supporting the conclusions of this article will be made available by the authors, without undue reservation.
